# Parental Educational Attainment and Social Environment of Urban Public Schools in the U.S.: Blacks’ Diminished Returns

**DOI:** 10.3390/children7050044

**Published:** 2020-05-10

**Authors:** Shanika Boyce, Mohsen Bazargan, Cleopatra H. Caldwell, Marc A. Zimmerman, Shervin Assari

**Affiliations:** 1 Departments of Pediatrics, College of Medicine, Charles R Drew University of Medicine and Science, Los Angeles, CA 90059, USA; ShanikaBoyce@cdrewu.edu (S.B.); Mohsenbazargan@cdrewu.edu (M.B.); 2 Departments of Family Medicine, University of California, Los Angeles (UCLA), Los Angeles, CA 90095, USA; 3 Department of Health Behavior and Health Education, School of Public Health, University of Michigan, Ann Arbor, MI 48109, USA; cleoc@umich.edu (C.H.C.); marcz@umich.edu (M.A.Z.); 4 Center for Research on Ethnicity, Culture, and Health (CRECH), School of Public Health, University of Michigan, Ann Arbor, MI 48109, USA; 5 Departments of Family Medicine, College of Medicine, Charles R Drew University of Medicine and Science, Los Angeles, CA 90059, USA

**Keywords:** youth, adolescents, population groups, ethnic groups, socioeconomic status, socioeconomic position, education, academic gap, school achievement, school environment

## Abstract

*Background:* Recent research has documented marginalization-related diminished returns (MDRs) of socioeconomic status (SES), defined as weaker effects of SES indicators, such as parental educational attainment, on securing tangible outcomes for the members of socially marginalized (e.g., racial and ethnic minority) groups, compared to privileged social groups (e.g., non-Hispanic Whites). *Aims:* To explore race/ethnic differences between non-Hispanic Blacks vs. non-Hispanic Whites who attend urban public schools on the effect of parental education on lower school environmental risk among American high schoolers. *Methods:* For this cross-sectional study, we borrowed the Education Longitudinal Study (ELS-2002) baseline data, a nationally representative study that enrolled 1706 10th grade youths who were attending urban public schools. From this number, 805 (47.2%) were non-Hispanic Black and 901 (52.8%) were non-Hispanic White youths. The dependent variable was the level of school social environmental risk measured using 18 items as self-reported, and was treated as a continuous variable. The independent variable was parental educational attainment, treated as a continuous measure. Gender, region, and parental marital status were the covariates. Race/ethnicity was the moderating variable. Linear regressions were applied to perform our data analysis. *Results:* Black students were found to attend schools with higher levels of social environmental risk. Youths with parents with a higher educational attainment were found to attend schools with a lower social environmental risk. We found a significant interaction between race (non-Hispanic Black vs. non-Hispanic White) and parental educational attainment on the level of school social environmental risk, suggesting that the protective effect of high parental education on reducing the school social environmental risk was smaller for non-Hispanic Black than for non-Hispanic White youths. *Conclusions:* Although high parental educational attainment is protective against social environmental risk for American youths, this protective effect is weaker for non-Hispanic Black than non-Hispanic White youths. The diminished returns of parental education in reducing school social environmental risk may explain why the effects of parental education on educational outcomes are smaller for non-Hispanic Black than non-Hispanic White youths (i.e., MDRs). The social environment indirectly generates racial youth educational disparities through deteriorating non-Hispanic Black youth educational outcomes across all SES levels. To prevent the confounding effects of private, suburban, rural, and Catholic schools, we limited this analysis to public urban schools. More research is needed on other settings.

## 1. Introduction

In the United States, there have been racial and ethnic gaps in academic achievement, with non-Hispanic Black students having less educational attainment compared to their non-Hispanic White counterparts [1,2]. Although the racial and ethnic achievement gaps have narrowed over the past 30–40 years, this disparity continues to exist [2]. This is problematic because there is a link between academic achievement/education and health, where poor academic achievement is linked to worse health outcomes across domains [3,4,5,6,7]. In terms of how differences in school quality between Black and White students affect health, a study by Frisvold and Golberstein showed a statistically significant reduction in racial gaps in disability associated with a convergence in school quality between Blacks and Whites [8]. 

Socioeconomic inequalities between non-Hispanic Black and non-Hispanic White families are believed to be a major contributing factor in explaining the Black–White achievement gap [2]. Socioeconomic differences by race/ethnicity, when assessing for educational achievement, tend to be reflected by parental educational attainment, as well as poverty status, income, marital status, and unemployment [2]. Several studies have shown that parental educational attainment is a more powerful predictor of youth academic achievement than other socioeconomic indicators such as income and poverty status [2,9,10]. At the same time, parental educational attainment is a core determinant of various aspects of health, such as smoking [11], mental well-being [12], alcohol binge drinking [13], subjective health [14,15], and chronic medical conditions [16]. It is important to note that a study by Masters, Link, and Phelan discusses the Fundamental Cause Theory, which makes “claims about the persistence of socioeconomic disparities in health”. The study tested the theory and found educational differences in U.S. adult mortality for preventable causes of death, whereby a higher education level was associated with lower mortality by preventable deaths. However, limitations were found to exist with this theory, as it did not provide explanations for gender and racial/ethnicity variations in health, education, and mortality risk [17].

The concept of marginalized-related diminished returns (MDRs) [18,19] refers to the decreased effect of SES indicators on health, development, and educational outcomes for racial/ethnic minority groups, compared to non-Hispanic Whites. This concept introduces an alternative explanation for understanding disparities. This framework is particularly useful to understand why racial and ethnic disparities exist across groups with a similar mean SES. According to the MDRs, similar resources (due to being of the same SES) do not translate into similar outcomes for minority groups compared to non-Hispanic Whites. This has been exhibited in several studies where non-Hispanic Black youths from high-income and highly educated families show poor health [15,20,21,22] compared to their non-Hispanic White counterparts. For example, middle-class non-Hispanic Black youths remain at risk of aggression [23], tobacco dependence [2,3], asthma [24], obesity [22,25], attention deficit hyperactivity disorder [26], chronic disease [16], depression [27], anxiety [28], and poor dental care [29]. This is similarly the case for non-Hispanic Black adults [30,31], Hispanic youths [32,33,34], Hispanic adults [35,36], and lesbian, gay, bisexual, and transgender (LGBT) individuals [37,38,39]. Research has also documented lower educational outcomes of middle-class Black youths compared to their non-Hispanic White counterparts [40,41,42]. 

It is important to note that other factors may be linked to Black–White disparities in academic achievement, such as differences in school quality and school resources [9]. Studies examining both factors have been inconclusive in showing that they contribute to racial and ethnic gaps in academic achievement [43,44,45,46]. Additionally, one study by Downey, Quinn, and Alcaraz found that there was no difference, when comparing schools serving poor and black children versus nonpoor white children, specific to the impact on reading scores [47]. 

Residential racial segregation still exists, the effects of which are felt most by middle-class Black families. Non-Hispanic Black families “do not have the same social, economic, and political advantages as middle-class non-Hispanic Whites” [48]. Additionally, differences in residential opportunities result in middle-class non-Hispanic Black families living in areas of concentrated poverty and their children attending “high poverty schools” compared to low-income non-Hispanic Whites [49]. Similar observations were discussed in the literature in 1999, when Williams discussed that racial differences in the quality of elementary and high school education were due to residential segregation. Williams highlighted that community resources (funds available at the local level) determine the quality of neighborhood schools, and thus residential segregation contributes to both concentrated poverty in residential areas and in the classroom [50]. It will therefore be important to know if the school environment (quality, risk, and resources) is an influential factor contributing to the racial/ethnic academic achievement gap. Furthermore, it remains to be seen if controlling for these factors will lead to the elimination of the achievement gap, especially in an environment where parental educational attainment between racial groups is similar. The persistence of this achievement gap after controlling for these factors would indicate that more upstream societal forces, such as racism and discrimination, or factors beyond schooling quality (such as labor market discrimination), are contributing to the problem. Currently, we are not aware of many previous studies that explore the diminished returns of parental education on school social environmental risk. 

Building on the prior research on MDRs (i.e., diminished returns of educational attainment and other SES indicators for marginalized, compared to mainstream, populations) [18,19] and borrowing data from a nationally representative study with extensive and rich data on school characteristics [51], we aim to compare racial/ethnic groups (non-Hispanic Black vs. non-Hispanic White), who attend urban public schools for the association between parental educational attainment and the risk of the school social environment, where the risk of the school social environment reflects behavioral and social aspects of the school. As explained above, this current study has the potential to extend the existing literature because it is one of the first to apply MDRs with school social environmental risk as the outcome. The contribution of this study will be to suggest that the social and behavioral school environment may have a role in explaining MDRs of parental education on educational outcomes of racial/ethnic minority groups (non-Hispanic Black vs. non-Hispanic White). As discussed by Urie Bronfenbrenner [52], the social environment has a large impact on all aspects of youth development, and students’ academic performance is not an exception to this rule. 

## 2. Methods

### 2.1. Design and Settings

This is a cross-sectional study. We conducted a secondary analysis of the Education Longitudinal Study (ELS) Wave 1 data [51]. Funded by the United States Department of Education (DOE), the ELS is a national study of educational outcomes of American 10th grade youths (ages 15–16). 

### 2.2. Sample and Sampling

In the Wave 1 data, the ELS study sample was youths who were in their 10th grade and were in a private, public, or Catholic school and in a rural, urban, or suburban school. The ELS applied a multi-stage stratified probability sampling strategy to recruit a nationally representative sample of 10th grade American youths. The analytical sample was 1706 youths.

The ELS Wave 1 sample is representative of United States youths at 10th grade. Although the ELS baseline included 10th graders from all race/ethnic groups, any student with mixed race, missing data on race, or a race other than non-Hispanic White or non-Hispanic Black was excluded. This included individuals who were Asian American, American Indians/Alaska Natives, mixed race, or unknown race/ethnicity. We also excluded Hispanic/Latino youth. Thus, we only included 1706 youths who were limited to non-Hispanic Black and non-Hispanic White youths.

### 2.3. Study Variables

The study variables were as follows: race and ethnicity as the moderators, parental education as the predictor, school social environmental risk as the dependent variable, and demographic factors (gender and family structure) and school characteristics covariates. 

#### 2.3.1. Race/Ethnicity 

Race (1 = Black versus 0 = White) and ethnicity (1 = Hispanic versus 0 = non-Hispanic) were both self-identified. Race/ethnicity was operationalized as a dichotomous variable: non-Hispanic Black = 1 and non-Hispanic White = 0.

#### 2.3.2. Parental Educational Attainment

Parental educational attainment was recorded as a continuous measure ranging between 1 and 8 with a higher score indicating a higher parental educational attainment. 

#### 2.3.3. Demographic Factors

Gender, region, and family structure were demographic covariates. Gender was 1 = male and 0 = female. Family structure was a dichotomous variable (1 = married, 0 = unmarried) and calculated based on the parents’ marital status. Region was a nominal variable: Northeast, Midwest, South, and West. The regions were entered into regressions as a series of indicator/dichotomous variables. 

#### 2.3.4. Outcome

Our dependent variable was a continuous score reflecting school social environmental risk. The following items were used to measure the outcome: (1) “How often are physical conflicts a problem at school?”, (2) “How often is robbery/theft a problem at school?”, (3) “How often is vandalism a problem at school?”, (4) “How often is the use of alcohol a problem at school?”, (5) “How often is the use of illegal drugs a problem at school?”, (6) “How often are students on drugs/alcohol at school a problem? ”, (7) “How often is the sale of drugs near school a problem?”, (8) “How often is the possession of weapons a problem at school?”, (9) “How often is the physical abuse of teachers a problem at school?”, (10) “How often is racial tension among students a problem at school?”, (11) “How often is student bullying a problem at school?”, (12) “How often is the verbal abuse of teachers a problem at school?”, (13) “How often is disorder in classrooms a problem at school?”, (14) “How often is student disrespect for teachers a problem at school?”, (15) “How often is gang activity a problem at school?”, “(16) How often are cult/extremist group activities a problem at school?”. These data only loaded on a single factor, according to our exploratory factor analysis. Responses were on five levels as (1) Never, (2) On occasion, (3) At least once a month, (4) At least once a week, and (5) Daily. We calculated a mean of all responses. The total score ranged from 1 to 5, with a higher score indicating a higher school social environmental risk (more behavioral and social problems at school). Cronbach Alpha = 0.867. 

### 2.4. Statistical Analysis

To analyze the ELS Wave 1 data, we used SPSS 23.0 (IBM Corporation, Armonk, NY) that enabled us to adjust for survey weights due to the multi-stage sampling design. Given that we adjusted for survey weights, the results are representative of the United States youth population. With a normally distributed outcome, we could perform linear regressions. We also did not find any evidence that was suggestive of multicollinearity. We tested and passed the assumption of homoscedasticity. We ran two linear regression models in the pooled sample. We also ran two race/ethnicity-specific models, one for non-Hispanic Whites and one for non-Hispanic Blacks. These models included race/ethnicity, gender, region, and parental marital status. From our linear regression models, we reported beta (b), 95% confidence interval, and *p*-value. *p*-values equal or less than 0.05 were considered as statistically significant.

### 2.5. Ethics

All youths gave assent. Their parents/guardians signed written consent. The Department of Education (DOE) institutional review board (IRB) approved the study protocol. Given that we used fully deidentified publicly available data, this analysis was exempted from a full IRB review.

## 3. Results

### 3.1. Univariate Analysis

Table 1 shows a summary of the descriptive statistics of our sample in the pooled sample as well as by race/ethnicity. This study included 1706 United States 10th grader youths who were attending urban public schools. This overall number was composed of 805 (47.2%) non-Hispanic Black and 901 (52.8%) non-Hispanic White youths. Non-Hispanic White and Non-Hispanic Black students differed in parental education and school social environmental risk.

### 3.2. Bivariate Analysis

Table 2 presents the summary of bivariate correlations between study variables in the pooled sample as well as by race/ethnicity. In the pooled sample, race/ethnicity was positively, and parental education was inversely, correlated with lower school social environmental risk. Within-group findings differ but the between-group contrast is only marginally significant.

### 3.3. Multivariable Analysis (Pooled Sample)

Table 3 presents the summary of two linear regression models of the pooled sample. In both models, race/ethnicity (non-Hispanic Black) and parental education were associated with school social environmental risk. The second model showed an interaction between race/ethnicity and parental educational attainment on school social environmental risk. This interaction term suggested that the effect of high parental education on reducing school social environmental risk is smaller for non-Hispanic Black than for non-Hispanic White youths (although this was only marginally significant). This finding suggests that non-Hispanic Black youths attend schools with a poor school environment, even when they have highly educated parents. This observation is an indicator of MDRs (diminished returns) of parental education for them. 

### 3.4. Multivariable Analysis (Racial/Ethnic Specific Models)

Table 4 presents the summary of two race/ethnic-specific linear regression models. In these models, parental educational attainment was associated with school social environmental risk. However, the association between parental education and school social environmental risk was present for non-Hispanic White youths only. In terms of significance, the returns were non-existent for non-Hispanic Black youths. 

## 4. Discussion

Overall, among those American youths who attend public urban schools, high parental educational attainment is associated with lower school risk/a better school environment. This association is weaker for non-Hispanic Black than for non-Hispanic White youths. That means non-Hispanic Black youths with highly educated parents would still attend high-risk schools, a level of risk which is unexpected and disproportionate given their parental educational attainment. This pattern is totally different than non-Hispanic White youths, for whom parental education drastically reduces school social environmental risk. 

Racial/ethnic, residential, and school segregation are major contributing factors behind the finding in our study that Black youths with highly educated parents attend high-risk schools and have a high level of school social environmental risk. Studies have shown that discrimination in the real estate industry and in the mortgage lending industry still exists today [45,46,48,49]. These practices lead to middle-class non-Hispanic Black families living in neighborhoods, that although improved socioeconomically compared to poorer co-ethnics, are not integrated with non-Hispanic White families of similar socioeconomic status [45]. As a result, where highly educated non-Hispanic Black families live in neighborhoods where schools are not socioeconomically integrated, there are high levels of concentrated poverty, and the quality of the schools are poorer compared to schools located in the residential neighborhoods in which non-Hispanic White middle-class families reside [45]. Such schools receive limited funding to provide resources in the form of courses, curriculum materials, equipment, and qualified teachers necessary to ensure that students have equal opportunities to be successful academically [49]. Additionally, a school environment that promotes academic excellence and a supportive teacher–student relationship are important factors that influence student engagement (positively through school adjustment and negatively through externalizing behavioral problems) and academic performance [50,51,52,53,54,55]. Thus, the environment created in schools with limited funding, to ensure the above factors are met and addressed, often leads to decreased academic outcomes and increased school social environmental risk observed among students.

While the residential levels of Black and White families may have a role, the results of this study may be more relevant to the policies that regulate how students are drawn to schools. That is, both high poverty neighborhoods and poor schools may have a role in explaining our findings. It is still unknown how the racial composition of a school influences race-related aggression and violence. While integration may have a healthy effect on increased school quality for Black youths, racially mixed schools may also contribute to racial tension. There is a need to study whether attending racially mixed schools correlates with more race-related aggression and violence or not.

The current study was on the social environment of school, mainly defined by peers and schoolmate risk behaviors. Peers, a major part of the social environment of students, have an important role on the social, developmental, and behavioral risk of youths. A report by Johnson showed that peer influences are major determinants of youth academic performance. Youths are strongly influenced by peer pressure on extracurricular behaviors such as drinking alcohol, smoking cigarettes, and using other illicit substances [56]. Still, we did not explicitly collect data on peers. 

Parental educational attainment mitigates some of the influence of the school social environment and peers on academic achievement [57,58,59]. We found that this protective effect, however, is diminished more for non-Hispanic Black than non-Hispanic White youths. Non-Hispanic Black youths still experience high-risk peers and social environments even when their parents have high education attainments. Thus, parental education does not extend to buffer the effect of peer pressure and a high-risk social environment for non-Hispanic Black youths. Chung showed that the educational background of a peer’s parents affects the educational outcomes of a child, however, their influence on drinking and risk behaviors is less clear [60,61]. Ultimately, parental educational attainment and that of peers’ parents may help to mitigate the influence of peers on academic performance, but not social behaviors. These findings suggest that middle-class non-Hispanic Black students that often attend schools with a poor school environment have a greater risk of engaging in high-risk social behaviors based on peer influence. 

Our results add to the growing literature on MDRs in the educational context that show a reduced likelihood of positive outcomes for marginalized people, even when they have access to resources.

### 4.1. Policy Implications 

It is very important that strong programs and policies are implemented to address the racial disparities that exist across SES levels. These policies must not only ensure equal access to resources, a problem experienced by low SES groups, but also address the broad societal and structural processes that uniquely place middle- to high-class non-Hispanic Black families at a relative disadvantage. These programs may help highly educated non-Hispanic Black families to secure tangible returns on their educational attainment. As discriminatory practices in the labor market are a suspected cause of MDRs, enforcement of the existing anti-discrimination laws may be needed to minimize the diminished returns of educational attainment experienced by non-Hispanic Black parents [26,62]. Additionally, increased efforts should be made to recognize and mitigate the societal and environmental barriers common in the daily lives of non-Hispanic Black families and within non-Hispanic Black communities. Furthermore, investments must be targeted towards urban public schools and educational programs for non-Hispanic Black and other minority youths to ensure equal school quality, resources, and a supportive environment, this includes improving disciplinary practices [63]. 

### 4.2. Limitations 

This study had several methodological limitations. Our cross-sectional design did not allow us to make conclusions with causal connections from the data. Additionally, the sample size across racial and ethnic groups was imbalanced, thus our race/ethnic-specific models may have different statistical power. Given that this study only included non-Hispanic Black and non-Hispanic Whites, future studies should include other ethnic minorities including Asians and Native Americans. The SES indicator assessed in this study was parental educational attainment. However, consideration should be given to assess other SES indicators such as wealth, income, employment, and area level SES in future studies. Despite the above limitations, the results of the study add to existing literature on MDRs and the racial achievement gap that persists in education. The strengths of the study are that the sample is random and the sample size is large and representative of the general population, which means the findings are generalizable. Additionally, this is the first study that tested MDRs of parental education on school risk. Further multi-level models should examine the effects of schools and the racial composition of schools.

## 5. Conclusions

In the United States, non-Hispanic Black youths still attend high-risk schools at all SES levels, given the diminished returns of their parental educational attainment. Such diminished returns of parental educational attainment result in a worse than expected behavioral and educational profile of non-Hispanic Black youths with high parental education. 

## Figures and Tables

**Table 1:** Descriptive statistics in the overall sample and by race/ethnicity (n = 1706).

	All		Non-Hispanic Whites		Non-Hispanic Blacks	
	n	%	n	%	n	%
Race						
White	901	52.8	901	10.0		
Black	805	47.2			805	10.0
Region *						
Northeast	163	9.6	52	5.8	111	13.8
Midwest	468	27.4	250	27.7	218	27.1
South	841	49.3	425	47.2	416	51.7
West	234	13.7	174	19.3	60	7.5
Gender						
Female	867	50.8	459	50.9	408	50.7
Male	839	49.2	442	49.1	397	49.3
	**Mean**	**SD**	**Mean**	**SD**	**Mean**	**SD**
Parental Education (1-8) *	4.54	1.99	4.83	2.02	4.22	1.92
School Environmental Risk (1-5) *	2.07	0.39	2.04	0.38	2.11	0.41

SD = Standard Deviation; * *p* < 0.05 for comparison of non-Hispanic White and non-Hispanic Black youths.

**Table 2:** Bivariate correlations of race, ethnicity, parental educational level, and school risk.

	1	2	3	4	5	6	7	8	9
**All**									
1 Race (non-Hispanic Black)	1.00	−0.17 **	0.14 **	−0.01	0.05	0.00	−0.34 **	−0.16 **	0.08 *
2 Region−West		1.00	−0.13 **	−0.25 **	−0.39 **	0.06 *	0.08 **	0.07 **	0.11 **
3 Region−Midwest			1.00	−0.20 **	−0.32 **	0.04	−0.03	−0.06 *	0.02
4 Region−Northeast				1.00	−0.61 **	−0.03	−0.04	−0.07 **	0.25 **
5 Region−South					1.00	−0.03	−0.00	0.04	−0.32 **
6 Gender (Male)						1.00	0.01	0.01	0.05
7 Parents Married/Live							1.00	0.12 **	−0.03
8 Parental Education								1.00	−0.14 **
9 School Risk									1.00
**Non-Hispanic Whites**									
1 Race (non-Hispanic Black)									
2 Region−West		1.00	−0.12 **	−0.30 **	−0.46 **	0.04	0.00	0.07 *	0.12 **
3 Region−Midwest			1.00	−0.15 **	−0.23 **	0.03	0.04	−0.08 *	−0.01
4 Region−Northeast				1.00	−0.59 **	0.01	0.01	−0.12 **	0.21 **
5 Region−South					1.00	−0.06	−0.03	0.09 **	−0.29 **
6 Gender (Male)						1.00	0.01	−0.03	0.09 *
7 Parents Married/Live							1.00	0.09 **	0.03
8 Parental Education								1.00	−0.17 **
9 School Risk									1.00
**Non-Hispanic Blacks**									
1 Race (non-Hispanic Black)									
2 Region−West		1.00	−0.11 **	−0.173 **	−0.29 **	0.09 *	0.08 *	0.01	0.18 **
3 Region−Midwest			1.00	−0.244 **	−0.41 **	0.04	−0.01	−0.00	0.04
4 Region−Northeast				1.00	−0.63 **	−0.07 *	−0.12 **	−0.01	0.31 **
5 Region−South					1.00	−0.01	0.07	0.01	−0.39 **
6 Gender (Male)						1.00	0.01	0.06	0.00
7 Parents Married/Live							1.00	0.07 *	−0.04
8 Parental Education								1.00	−0.06
9 School Risk									1.00

* *p* < 0.05 (Spearman); ** *p* < 0.01 (Spearman).

**Table 3:** Summary of linear regressions on the interactive effects of race/ethnicity and parental educational attainment on school risk score.

	Model 1 (Main Effects)					Model 2 (Interaction Effects)				
	b	SE	95% CI		*p*	b	SE	95% CI		*p*
Race (non-Hispanic Black)	0.09	0.03	0.04	0.15	<0.001	−0.02	0.06	−0.14	0.10	0.755
Region										
Northeast	−0.22	0.05	−0.32	−0.12	<0.001	−0.22	0.05	−0.33	−0.12	<0.001
Midwest	−0.03	0.04	−0.11	0.04	0.421	−0.03	0.04	−0.11	0.04	0.392
South	−0.29	0.04	−0.36	−0.22	<0.001	−0.29	0.04	−0.36	−0.22	<0.001
Gender (Male)	0.04	0.02	0.00	0.09	0.074	0.04	0.02	−0.01	0.09	0.081
Parents Married/Live	0.00	0.03	−0.05	0.05	0.885	0.00	0.03	−0.05	0.05	0.885
Parental Education	−0.02	0.01	−0.03	−0.01	0.001	−0.03	0.01	−0.05	−0.01	<0.001
Parental Education x Race						0.02	0.01	0.00	0.05	0.050
Intercept	2.27	0.05	2.18	2.37	<0.001	2.32	0.06	2.21	2.43	<0.001

b = regression coefficient; SE = standard error; CI = Confidence Interval.

**Table 4:** Summary of two series of linear regressions on the effects of parental educational attainment on school risk.

	Model 3 (Non-Hispanic Whites)					Model 4 (Non-Hispanic Blacks)				
	b	SE	95% CI		*p*	b	SE	95% CI		*p*
Region										
Northeast	−0.18	0.08	−0.33	−0.03	0.017	−0.44	0.08	−0.60	−0.27	<0.001
Midwest	−0.03	0.04	−0.11	0.06	0.557	−0.18	0.08	−0.33	−0.03	0.021
South	−0.18	0.04	−0.26	−0.10	<0.001	−0.57	0.07	−0.71	−0.42	<0.001
Gender (male)	0.06	0.03	0.00	0.12	0.046	0.01	0.04	−0.07	0.08	0.886
Parents Married	0.02	0.03	−0.04	0.08	0.492	−0.03	0.04	−0.11	0.05	0.436
Parental Education	−0.03	0.01	−0.05	−0.02	<0.001	−0.01	0.01	−0.03	0.01	0.565
Intercept	20.26	0.06	20.14	20.37	<0.001	20.55	0.09	20.38	20.72	<0.001

b = regression coefficient; SE = standard error; CI = Confidence Interval.
